# A promising and cost-effective biochar adsorbent derived from jujube pit for the removal of Pb(II) from aqueous solution

**DOI:** 10.1038/s41598-020-64191-1

**Published:** 2020-05-04

**Authors:** Junkai Gao, Yu Liu, Xuebin Li, Mouyuan Yang, Jinbao Wang, Yan Chen

**Affiliations:** School of Port and Transportation Engineering, Zhejiang Ocean University, Zhoushan, 316022 China

**Keywords:** Pollution remediation, Environmental impact

## Abstract

This study evaluated the Pb(II) sorption capacity of jujube pit biochar (JPB) in aqueous solution, which was derived from jujube pit by pyrolysis and used as a promising and economical adsorbent. More importantly, the utilization of JPB could realize the recycling of agricultural residues. The JPB was characterized using conventional science technologies, including SEM, BET and FT-IR, and the sorption capacity of JPB for lead ions was investigated according to different adsorption parameters, such as the kinetics data, solution pH, isotherms data, coexisting ions of Na^+^ and K^+^, desorption and reusability, and solution temperature. The results of kinetics data suggested that the lead ion adsorption process by JPB could be fast to reach equilibrium within 30 min. Additionally, the adsorption capacity of JPB for Pb(II) was calculated to be maximum for 137.1 mg/g at pH 6.0. More importantly, after five cycles of desorption and reuse, the JPB still reached 70% of its original adsorption capacity. All the results suggested that JPB had a broad application prospect for the purification of lead ions in practical.

## Introduction

Pb(II) has serious contamination with toxicity in aqueous soulution^1^ that is extremely harmful to human health^2–4^. Removing lead ions from wastewater has become an important task for humans. To date, some physical and chemical technologies, such as electrodialysis^5^, flocculation^6^, ion exchange^7^, chemical precipitation^8^, membrane filtration^9^ biosorption^10^ and adsorption^11^, have been applied to remove lead ions. Among all these purification strategies, adsorption is a promising and attractive method because the operation process for adsorption is simple, the operation cost is low and the possibility of secondary pollution is minimal^12^. According to previous research, different types of adsorbents, such as montmorillonite^13^, bionanocomposite^14^, activated carbon^15^, activated carbon fiber^16^, coal^17^, periwinkle shells^18^, and nanocomposite^19^, have been used for metal ions adsorption. The conventional adsorbent material, activated carbon, is widely used in wastewater purification and has attracted remarkable attention for considerable adsorption capacity and complex pore structure. However, traditional activated carbons have some defects, namely, a high fabrication cost and low reusability^20^. Therefore, research on carbon-based adsorption materials with high adsorption performance and low cost is of great importance and practical application value.

Biochar is a carbon material with abundant pores that is derived from biomass and prepared by pyrolysis. The merits of considerable adsorption capacity, a low preparation cost and an easy preparation process have made biochar adsorbents a current research focus^21^, and various kinds of biochar have been used as adsorption materials to remove Pb(II). Lee et al. measured the sorption capacity of GB (gingko leaf biochar), PB (peanut shell biochar) and MB (Metasequoia leaf biochar) for lead ions, and the results showed that the adsorption capacities of lead ions by GB, MB, and PB were 138.89, 34.01, and 30.67 mg/g, respectively^1^. Shen *et al*. studied the adsorption capacity of Pb(II) by rice straw biochars that were prepared by using the pyrolysis method at 300 °C, 500 °C and 700 °C, and the adsorption capacities were 109.59, 153.81 and 171.34 mg/g, respectively^22^.

Jujube is distributed in subtropical and temperate regions and possesses value as a food and nutriment due to its therapeutic properties^23,24^. Jujube pit is considered a byproduct of agricultural waste and is usually thrown away directly after the jujube flesh is extracted. Jujube pits are abundant and easy to collect in China and have several excellent characteristics for clear natural structure, low ash content and hardened lignocellulosic composition, which make jujube pits become a reasonable raw material for the preparation of biochar^25,26^.

**Figure 1:**
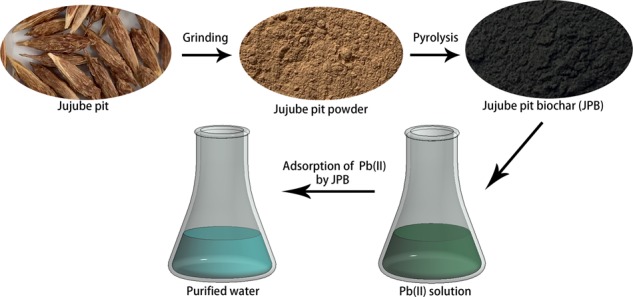
Preparation and utilization procedure of JPB.

In the present work, JPB was synthesized using jujube pit as the raw material by a simple pyrolysis method, which has the merits of cost effectiveness and ease of operation. The preparation and utilization procedure of JPB was shown in Fig. 1. Because the JPB was derived from a byproduct of agriculture, the utilization of JPB could realize the recycling of agricultural residues. The properties of the prepared JPB were characterized by SEM for surface structure, FT-IR for basic chemical groups, and BET for pore distribution and particle diameter size. The adsorption behavior of lead ions by JPB was studied including contact time, lead ion concentration, pH, coexisting ions and solution temperature, respectively. In addition, the reusability of JPB for lead ion removal was investigated in order to estimate its adsorption capacity after several cycles. According to the experimental results, the JPB usded as a novel adsorbent for lead ion adsorption was favorable, and therefore, JPB has the potential to be a promising and cost-effective biochar adsorbent for removing lead ions in practical applications.

## Materials and methods

### Experimental materials

The jujube pits were obtained from jujubes obtained from Hetian, Xinjiang, China. Lead nitrate used for the preparation of lead ion solutions was produced by Sinopharm Chemical Reagent Co. Ltd. L-Ascorbic acid was bought from Sinopharm Group Chemical Reagent Co. Ltd. Potassium ferrocyanide trihydrate and ammonium fluoride were purchased from Shanghai Macklin Biochemical Co. Ltd. Thymol blue and xylenol orange tetrasodium salt were obtained from Aladdin Industrial Corporation, and sodium acetate buffer solution was purchased from Sigma-Aldrich.

### Preparation of jujube pit biochar

JPB was synthesized using jujube pit as the raw material. First, the residual pulp impurity on the surface of the jujube pit was removed by brushing. Then, the jujube pit was washed for its surface residues with distilled water and then dried at 105 °C for a whole day (24 hrs). After that, the clean and dry jujube pits were broken into powder by a high-speed disintegrating machine (Zhejiang Hongjingtian Industry and Trade Co. Ltd), and then the powder was sieved through a standard sample sieve (Taizhou Yueyang Trading Co. Ltd) with a size of 200 mesh per inch. The selected jujube pit powder was pyrolyzed at 800 °C for two hours under a nitrogen atmosphere in a tubular furnace to obtain JPB.

### Lead ion adsorption

In batch sorption experiments, the room temperature was approximately 298 K, and the solution pH was 6, which was adjusted by 0.1 M HNO_3_ or NaOH. A total of 0.01 g JPB powder was added to 25 mL lead ion solution for mixture, and then the mixture was placed into a shaker (THZ-98A, Shanghai Yiheng Scientific Instrument Co., LTD, China) and stirred for a certain time. After that, the mixture was removed and then separated by a vacuum pump for filtration. Each experiment was carried out using two parallel samples, and if the error between the test results of the two parallel samples was less than five percent, the test results would be adopted. While the whole adsorption process was completed, the concentration of lead ions in the solution was measured by a visible spectrophotometer (723), which was made by Shanghai Jinghua Technology Co., LTD.

The adsorption quantity of lead ions by JPB was obtained according to the adsorption balance Eqs. (1) and (2), where q_t_ (mg/g) is the quantity of lead ions adsorbed by JPB when the experiments are conducted for t min and q_e_ (mg/g) is the equilibrium adsorption capacity.

1 $${q}_{t}=\frac{({c}_{0}-{c}_{t})\cdot v}{m}$$

2 $${q}_{e}=\frac{({c}_{0}-{c}_{e})\cdot v}{m}$$

In Eqs. (1) and (2), C_0_, C_e_, and C_t_ (mg/L) are the concentration of lead ions in solution at the beginning, time t, and equilibrium, respectively; V is the volume of lead ions in solution (L); and m is the dry JPB quantity (g). Each adsorption experiment was performed twice, and the experimental data are reported as average values.

### Effect of Na^+^ and K^+^

In batch sorption experiments, the solution temperature and the pH were 298 K and 6.0, and meanwhile, NaNO_3_ was used as the presence of Na^+^ and KNO_3_ was used as the presence of K^+^, respectively. Additionally, the concentration of Na^+^ or K^+^ ions were set up for 0, 0.1, 0.2, 0.3, 0.4 and 0.5 mol/L. In these experiments, the concentration of prepared Pb(II) solution was 150 mg/L.

### Effect of temperature

The operating temperature was in a range from 298 K to 308 K, and the solution pH was 6. The prepared Pb(II) concentration for the batch experiments was 150 mg/L, and the adsorbent dosage was 400 mg/L.

### Desorption and reusability experiments

The reusability experiments were conducted by placing 10 mg JPB powder in a conical flask mixed with 25 ml lead ion solution (100 mg/L); the temperature and pH were 298 K and 6.0, respectively. Then, the mixture containing JPB adsorbent and lead ions was stirred in a shaker at 200 rpm for 90 min. After that, the mixture was filtered to separate the JPB adsorbent, the lead ion concentration in the filtered solution was tested, and then the adsorption quantity of lead ions by JPB could be calculated. Then, the spent JPB samples were regenerated using 0.2 M hydrochloric acid and washed using distilled water followed by drying^26^, after which the regenerated JPB was used in the following adsorption cycle.

### Characterization of raw material (jujube pit) and biochar (JPB)

Surface morphological images of JPB particles were detected using a scanning electron microscope (SEM, FEG-250, FEI, USA). The chemical structures of jujube pit powder and JPB were both analyzed by Fourier transform infrared spectroscopy (FTIR, VECTOR22, Bruker, Germany). The Brunauer-Emmett-Teller (BET, NOVA 2000e, Quantachrome, America) method was used for accurate calculation of the specific surface area of JPB, and the pore size distribution was specifically analyzed using the BJH method based on the nitrogen adsorption-desorption isotherms. Before the nitrogen adsorption-desorption process, JPB powder samples were dried at 323 K, and 0.1 g samples were added into the sample tube, which was then putted into the degassing station to degas, and the degassing temperature was 77.3 K.

## Results and discussion

### Characterization of JPB

**Figure 2:**
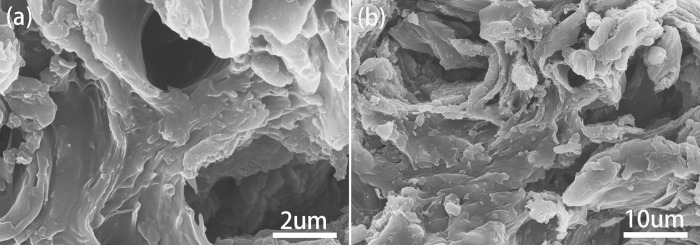
SEM images of JPB.

SEM images of the jujube pit biochar are shown in Fig. 2. Several distinct characteristics were apparent on the JPB surface. A plenty of clear irregular wrinkles were covered on the JPB surface, while a lamellar nature and significant roughness were also observed from the images. Additionally, carbon-based functional groups contribute to the roughness on the surface of JPB^4^. Such surface morphology structures possess many advantages^27^. For example, the wrinkled surface of JPB could make its surface area enlarged and make more active and adsorbable sites exposed on the outside^28^, which could make a promotion for lead ions adsorbed onto the JPB surface more easily^29^.

**Figure 3:**
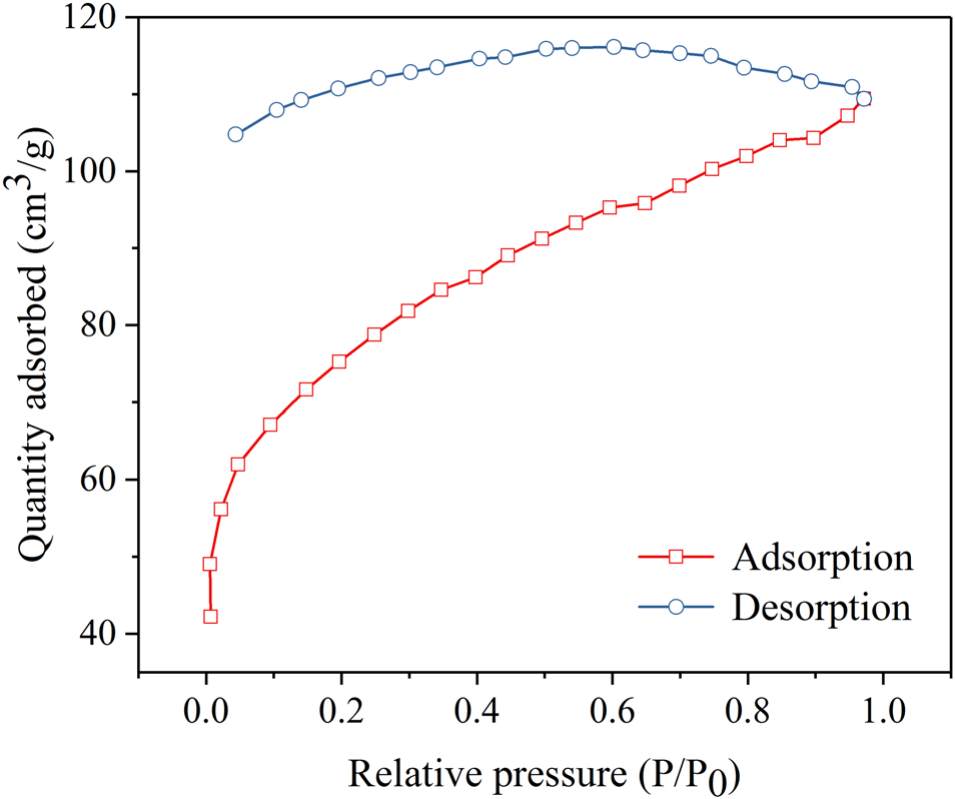
Nitrogen adsorption/desorption isotherms of JPB.

**Figure 4:**
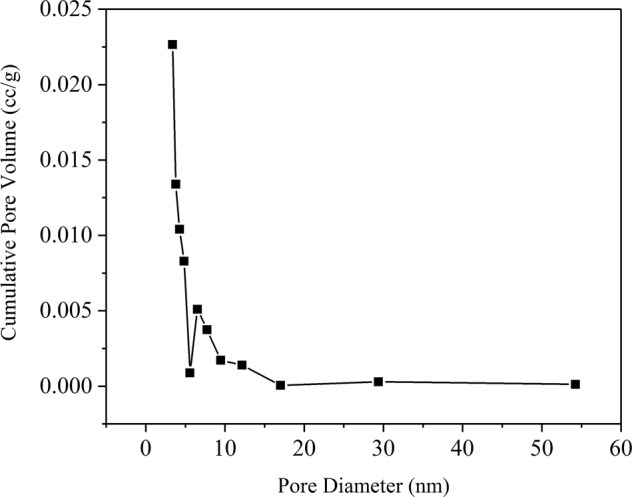
Pore size distribution of JPB.

The curve of nitrogen adsorption-desorption isotherms is presented in Fig. 3. The JPB possessed a lot of small dispersed pores, which caused some nitrogen to leave in the sample pores during desorption contributing to the lack of closure in the adsorption/desorption isotherm^30^. And the graph of pore size distribution of JPB are presented in Fig. 4., respectively. The average pore diameter and surface area of JPB were 3.387 nm and 246.9 m^2^/g, respectively, while the test result for pore volume was tested to be 0.054 m^3^/g. The large surface area could contribute to interactions between the adsorption sites of JPB and lead ions^31^. Moreover, the pores were large enough for lead ions to enter the inner surface of JPB, and hence, its adsorption capacity could be enhanced^31^.

**Figure 5:**
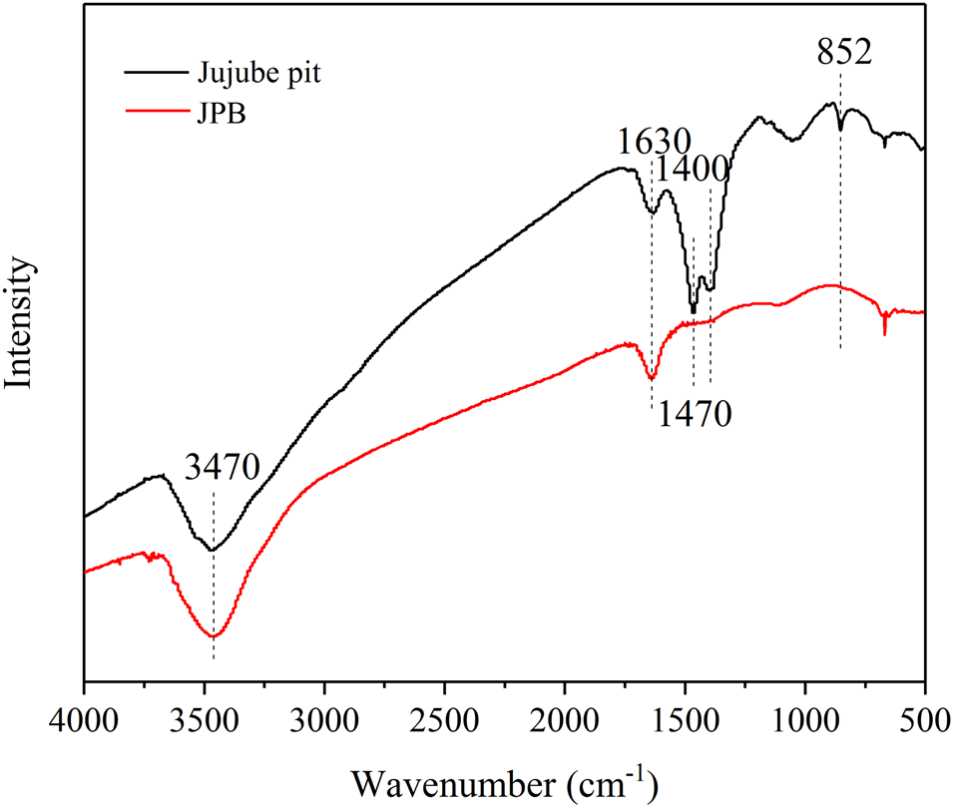
FT-IR spectra of jujube pit and JPB.

The FTIR spectra of the JPB and the raw material (jujube pit) are shown in Fig. 5. In these spectra, the intensity of the peaks at 852, 1400, and 1470 cm^−1^ for JPB decreased and the intensity of the peaks at 1630 and 3470 cm^−1^ increased after pyrolysis. The band at 852 cm^−1^ could be ascribed to the weak stretching vibration of aromatic C-C^22,32^. In the jujube pit spectrum, the bands observed at 1400 cm^−1^ and 1470 cm^−1^ originated from the strong stretching vibration of C-H in alkanes or alkyl groups and C=O in carboxyl groups, respectively. After pyrolysis, the two peaks disappeared in the spectrum of JPB, which was attributed to the decomposition of cellulose and hemicellulose^22^. Moreover, the peak detected at approximately 1630 cm^−1^ resulted from the stretching vibration of C=C^25^. The peak at 3470 cm^−1^ was assigned to hydroxide (-OH) stretching, and the hydroxyl groups were mainly from carboxylic acids and some of the water in the detected samples^33^.

### Sorption kinetics

**Figure 6:**
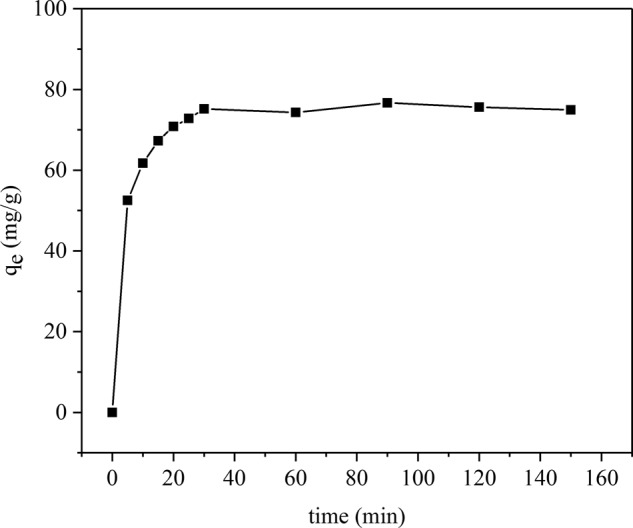
Effect of contact time on Pb(II) adsorption on JPB.

Contact time is a significant parameter for evaluating sorption kinetics in adsorption processes. The change in the adsorption amount of Pb(II) on JPB is depicted in Fig. 6. In this experiment, the concentration of Pb(II) was 150 mg/L, and the solution pH was set to 6. The adsorption process could reach equilibrium after 30 minutes of reaction, and it could be divided into three different stages for analysis. In the first stage, the adsorption capacity of Pb(II) by JPB increased rapidly in the initial fifteen minutes, and the adsorption amount reached 62.5 mg/g, which was 80% of the saturated adsorption capacity. The rapidly increasing adsorption amount for the first period was primarily owing to the abundant vacant sites exposed on biochar, which could contribute to making the lead ions attached onto the surface of JPB easily^4^. Moreover, the higher concentration gradient made Pb(II) rapidly adsorbed by the JPB. The second stage was the next 15 min; the adsorption capacity could still increase, but the increasing speed became much slower than that during the first stage, and the adsorption capacity gradually became close to the saturated value. In the last stage, the adsorption amount was maintained at a relatively stable value, which suggested that the adsorption equilibrium was reached and the amount would not change with the reacting time changing. Based on the study results, the adsorption equilibrium of Pb(II) on JPB could be achieved sufficiently in 30 min.

Three classical kinetics models were utilized to study the adsorption procedure and rate of JPB for Pb(II) removal, including pseudo-first-order^34^, pseudo-second-order^35^ and Elovich kinetic equations^25^, which can be presented as:

3 $$ln({q}_{e}-{q}_{t})=\,ln\,{q}_{e}-{k}_{1}\cdot t$$

4 $$\frac{t}{{q}_{t}}=\frac{1}{{k}_{2}{q}_{e}^{2}}+\frac{t}{{q}_{e}}$$

5 $${q}_{e}=\frac{1}{\beta }{lnt}+\frac{1}{\beta }\,{ln}(\alpha \beta )$$

where the adsorption rate constant of pseudo-first-order model is represented as k_1_ (min^−1^), the adsorption rate constant of pseudo-second-order is represented as k_2_ (g/(mg·min)), q_t_ is the lead ion adsorption quantity after the experiment is conducted for t min and q_e_ is the amount when the adsorption experiment reaches equilibrium, α is the initial adsorption rate (mg/(g min)) and β is a constants for desorption constant (g/mg).

**Figure 7:**
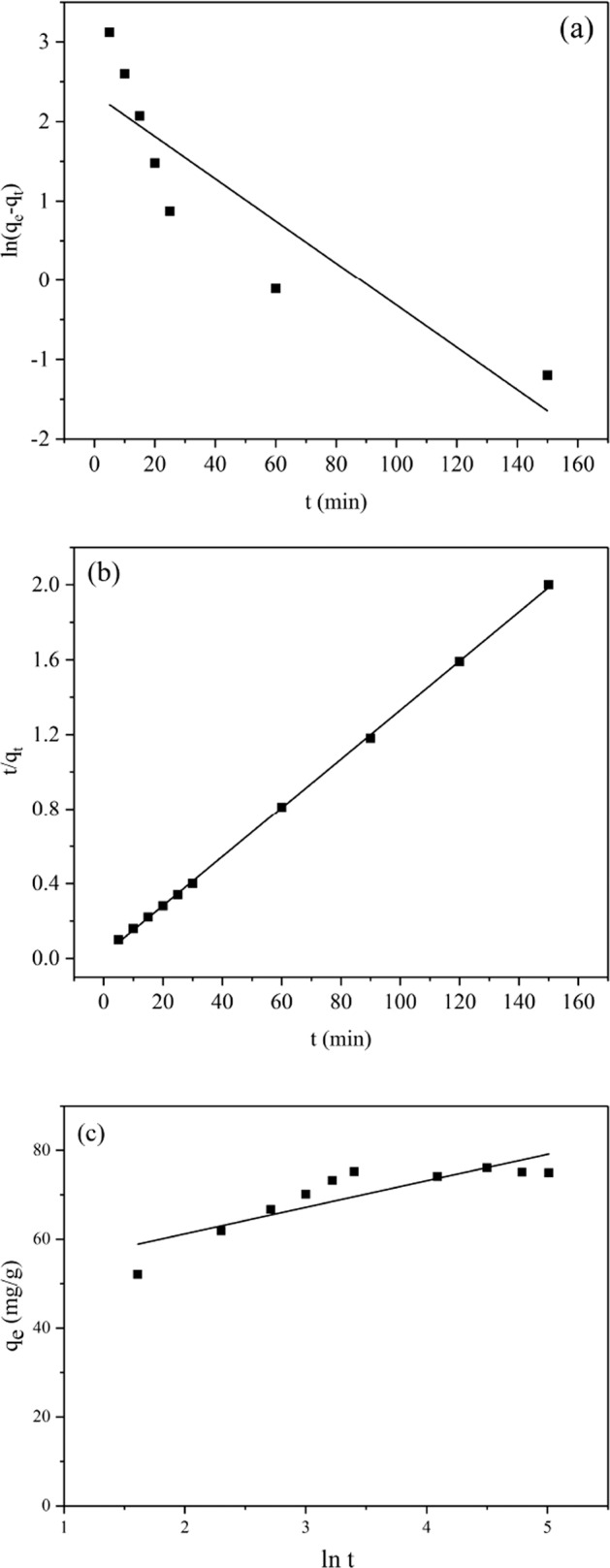
(**a**) Pseudo-first-order kinetic model, (**b**) pseudo-second-order kinetic model and (**c**) Elovich kinetic model for Pb(II) adsorption on JPB.

The linear relationship of the kinetic models was shown in Fig. 7. The relevant parameters of the kinetic equations were exhibited in Table 1. By analyzing and comparing the experimental data in Table 1, an unreasonable experimental adsorption capacity q_e_ was predicted by pseudo-first-order kinetic and Elocich kinetic model due to the significantly lower R^2^ value, indicating that these models provided an improper fit to the sorption kinetics. In contrast, the pseudo-second-order model with a correlation coefficient (R^2^) approaching to 1 was proved to be more appropriate to fit the kinetics model, and the calculated value of q_e_ (83.3 mg/g) was closer to the actual adsorption capacity (75.2 mg/g) obtained from the experiment than that calculated from the pseudo-first-order model. Therefore, the applicability to the adsorption process of Pb(II) on JPB was better to be described by pseudo-second-order model.

**Table 1:** Relevant adsorption parameters kinetic models.

Model	Parameter	Value
Pseudo-first-order	K_1_ (min^−1^)	0.026
	q_e_ (mg/g)	10.38
	R^2^	0.800
Pseudo-second-order	K_2_ (g/(mg·min))	0.04
	q_e_ (mg/g)	83.3
	R^2^	0.999
Elovich	α(mg/(g min))	143.1
	β(mg/g)	0.077
	R^2^	0.71

### Adsorption isotherm

**Figure 8:**
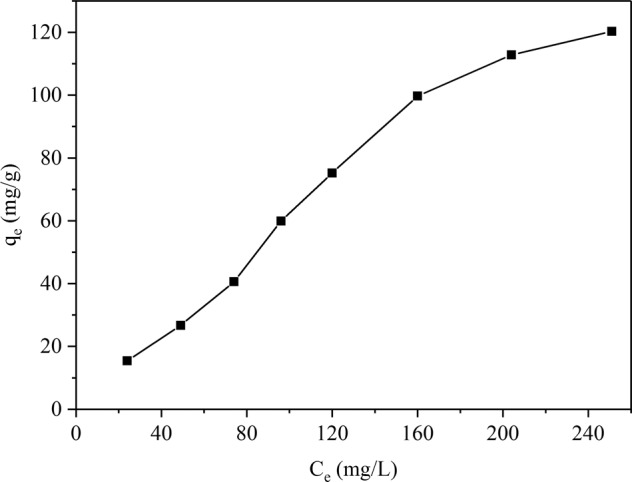
Sorption isotherms of Pb(II) on JPB.

The adsorption amount of the adsorbent for various concentrations at a fixed temperature is shown by the adsorption isotherm. Relevant experiments were conducted with ambient temperature for 298 K, meanwhile the Pb(II) concentrations ranged from 30 to 150 mg/L and the solution was in the condition of faintly acid for the pH was 6.0. The adsorption result is displayed in Fig. 8. The data revealed that as the concentration of Pb(II) increased, the increased rate of adsorption amount slowed down gradually, progressively saturating the adsorbent^36^.

Three different isotherm models (Langmuir, Freundlich and Tempkin) were used to study the maximum adsorption capacity, and the models are expressed as Eqs. (6–8), respectively^25,37,38^:

6 $$\frac{{C}_{e}}{{q}_{e}}=\frac{1+{C}_{e}{K}_{L}}{{q}_{m}{K}_{L}}$$

7 $${ln}\,{q}_{e}=\,{ln}\,{K}_{f}+\frac{1}{n}\,{ln}\,{C}_{e}$$

where q_e_ represents the equilibrium adsorption capacity of lead ions adsorbed by a unit mass of JPB adsorbent, q_m_ represents the maximum mass of lead ions adsorbed by a unit mass of JPB adsorbent, C_e_ represents the concentration of Pb(II) at the time of equilibrium, and K_f_ and n are two other constants.

8 $${q}_{e}=\frac{{RT}}{{bT}}\,{ln}\,{C}_{e}+\frac{{RT}}{{bT}}\,{ln}\,{K}_{T}$$

where B_T_ = RT/b_T_ (J/mol) is the Temkin constant, K_T_ (L/g) is the adsorption capacity and R (8.314 J/mol K) is the universal constant and T (K) is the absolute temperature.

**Figure 9:**
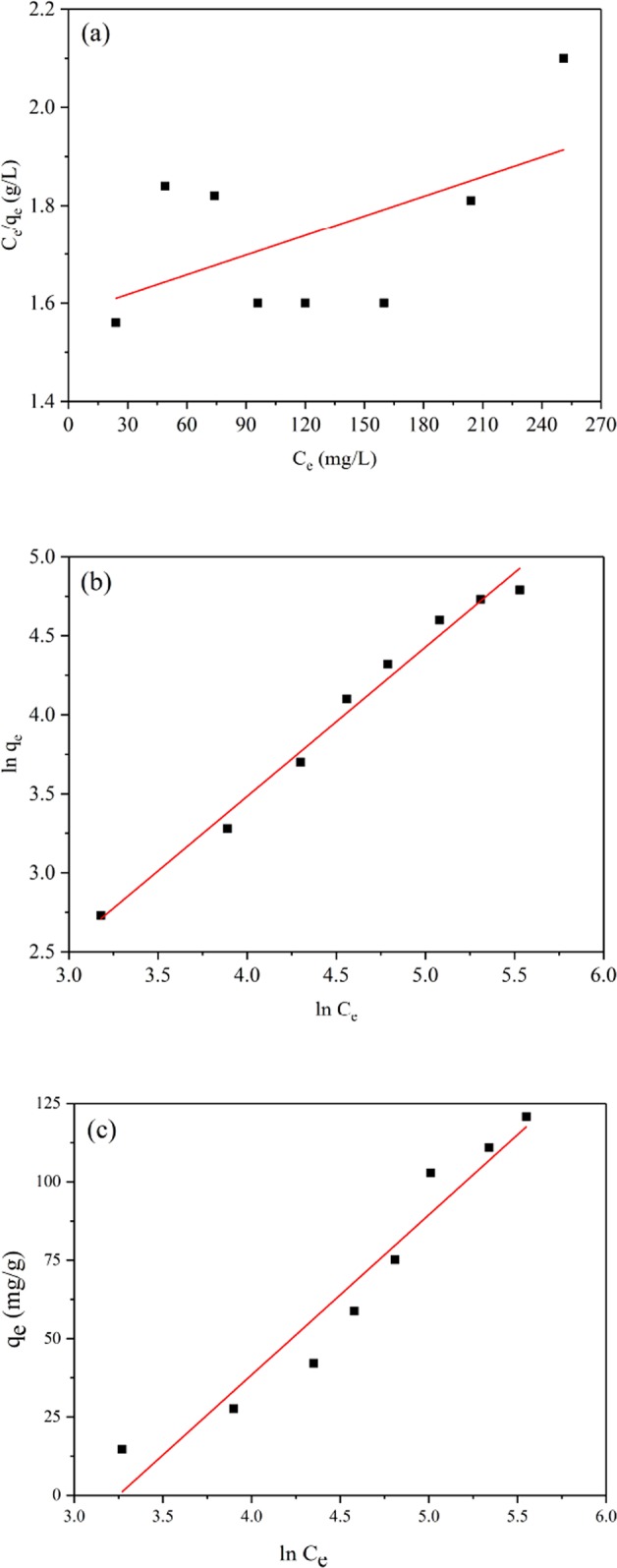
Langmuir isotherm model plot (**a**), Freundlich isotherm model plot (**b**) and Tempkin isotherm model plot **(c**) for Pb(II) sorption on JPB.

The fitted curves according to the three classic models are presented in forms of linear relationship in Fig. 9., and relevant experimental data obtained from the adsorption isotherms are exhibited in Table 2. Comparing the coefficients demonstrated that the Freundlich model (R^2^ = 0.987) could describe the applicability to the isotherm better than the Langmuir model (R^2^ = 0.317) and the Tempkin model (R^2^ = 0.9249), and the calculated adsorption capacity using the Freundlich model (137.1 mg/g) was approaching to the experimental amount obtained from the experiment (120.7 mg/g) fairly. Additionally, the Freundlich equation suggested that the process of lead ion adsorption on JPB was multilayer adsorption in a heterogeneous system^36,39^. More importantly, the comparison between the adsorption capacities of JPB and other adsorbents was exhibited in Table 3, which suggested that the performance of JPB was favourable and competitive, and therefore, the JPB developed in this study had a broad application prospect for the purification of lead ions.

**Table 2:** The parameters of the adsorption isotherms of Pb(II) adsorption on JPB.

Model	Parameter	Value
Langmuir	K_L_	0.003629
	q_m_ (mg/g)	192.0
	R^2^	0.3172
Freundlich	K_F_	0.7460
	q_m_ (mg/g)	137.1
	R^2^	0.9846
Tempkin	K_T_ (L/mg)	0.0592
	B_T_	33.71
	R^2^	0.9249

**Table 3:** Comparison of adsorption capacities between JPB and other adsorbents.

Sample	q_m_ (mg/g)	Contacting time (min)	Reference
JPB	137.1	30	Present work
WB^a^	87	60	^46^
GB^b^	138.9	40	^1^
RSB500^c^	144.3	360	^22^
RPK^d^	128.1	470	^25^
CABCU^e^	86.9	20	^47^

^a^WB: wheat bran. ^b^RSB500: rice straw biochar. ^c^RPK: date palm rachis. ^d^GB: gingko leaf biochar. ^e^CABCU: calcium alginate beads of caryota urens.

### Effect of pH

**Figure 10:**
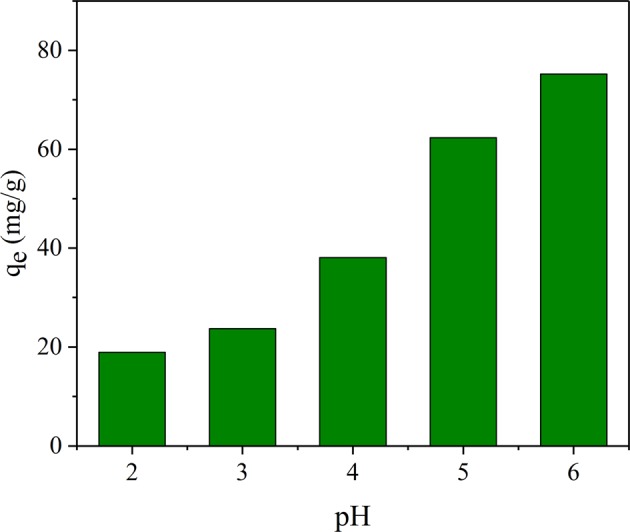
The capacity of lead ions for pH.

The pH is a significant adsorption parameter that has several distinct impacts on the efficience of metal ions removal, and since a precipitate is formed when Pb(II) is under alkaline conditions, a series of experiments were carried out in acidic conditions at 298 K. In addition, the solution pH was adjusted accurately by HNO_3_ solution (0.1 M) and NaOH solution (0.1 M), and the used dosage of JPB adsorbent was 0.4 g/L. The adsorption amounts for different solution pH values are presented in Fig. 10., and the results revealed that the adsorption amount of lead ions on JPB increased with the pH increased from 2.0 to 6.0. When the pH was lower, the solution contained a large amount of hydrogen ion (H^+^) and hydronium ion (H_3_O^+^) groups, and these groups competed for the fixed adsorption sites with Pb(II), resulting in fewer lead ions being adsorbed on the JPB. Moreover, because a repulsion effect occurs between cationic compounds, the hydrogen ion (H^+^) and hydronium ion (H_3_O^+^) groups distributed on the JPB surface would repulse the lead ions and make it away from the JPB surface^40^. Hence, Pb(II) adsorption on JPB was restricted in strengthened acidic condition. With the acid of solution condition weakened, the concentration of hydrogen ions (H^+^) and hydronium ions (H_3_O^+^) decreased, and more suitable adsorption sites were exposed to lead ions, leading to improved lead ion adsorption effectiveness by JPB.

### Effect of Na^+^ and K^+^

**Figure 11:**
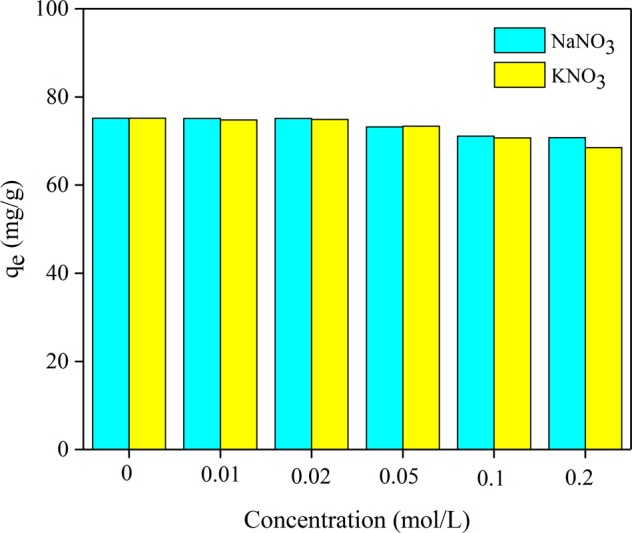
The adsorption of lead ions for JPB with Na/K ions.

An investigation for the impacts of coexisting ions on lead ions adsorption was taken in this section. The examined data is available in Fig. 11., and the result showed that the Na^+^ and K^+^ had a slight influence of inhibition on the adsorption of lead ions, and the adsorption capacity of JPB for lead ions was slightly decreased with increasing the concentrations of coexisting ions; the slight inhibitory effect might be due to the competition of positive ions, Na^+^ (K^+^) and Pb^2+^, for the active adsorption sites during the adsorption process^11,41^. However, divalent alkaline metal ions (Pb^2+^) were favored over monovalent alkali metal ions (Na^+^ and K^+^) in competition for the binding sites of JPB^36^. Therefore, the JPB developed in this study has great potential for actual applications.

### Effect of temperature and adsorption thermodynamics

**Figure 12:**
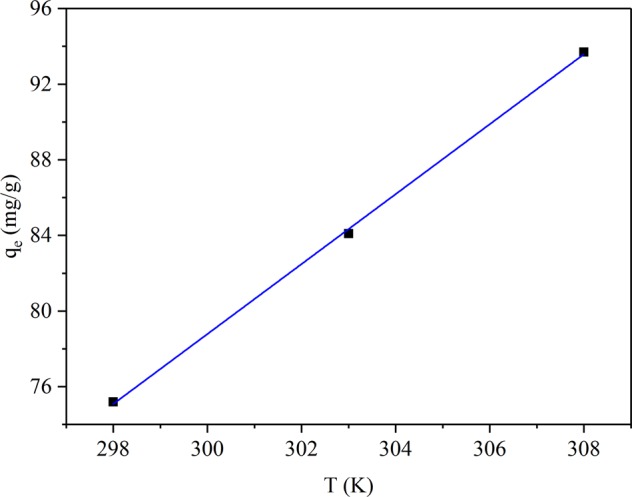
Effect of temperature on Pb(II) removal by JPB.

**Figure 13:**
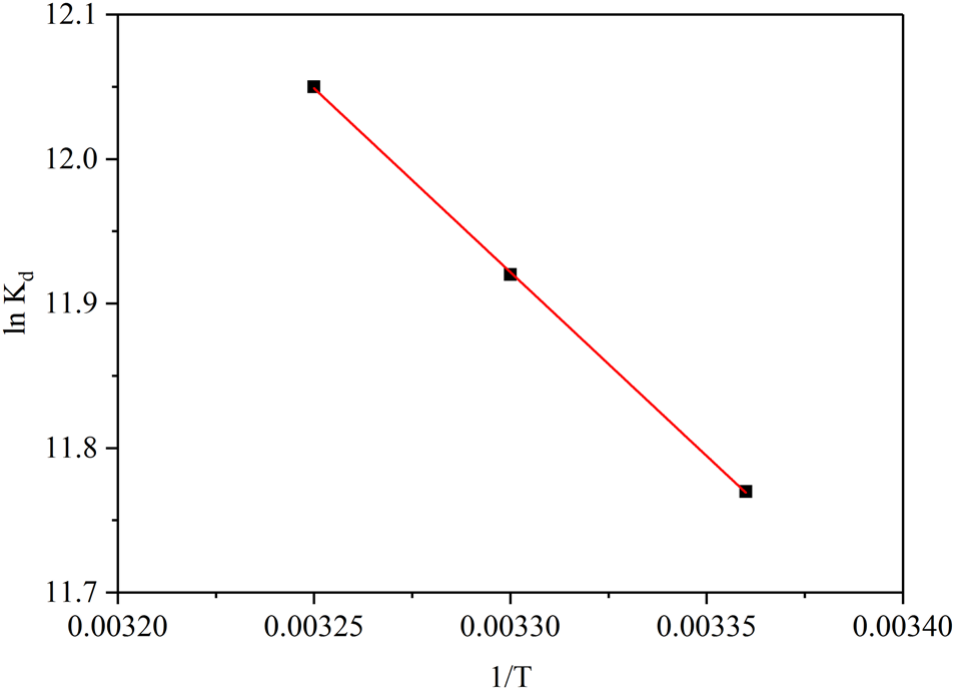
The linear graph of lnK_d_ versus 1/T for the adsorption of Pb(II) on JPB.

The relation of lead ion adsorption capacity by JPB for different temperatures in aqueous solution was investigated. The results of sorption capacity at different temperatures are exhibited in Fig. 12., and the adsorption capacity was approximately linear with temperature. The Pb(II) adsorption capacity on JPB was improved with the increase of temperature, which confirmed that the adsorption process of Pb(II) on JPB was endothermic. The adsorption thermodynamic parameters, such as free energy (∆G), enthalpy (∆H) and entropy (∆S), were obtained according to Eq. (9)^42^.

9 $${ln}\,{K}_{d}=\frac{\varDelta S}{R}-\frac{\varDelta H}{{RT}}$$

where K_d_ refers to the adsorption distribution coefficient, T represents temperature (K), and R is the universal gas constant. The thermodynamic parameters ∆H and ∆S were determined according to the linear relation between ln K_d_ and 1/T (Fig. 13.). The value of ∆G was obtained from Eq. (10)^42^.

10 $$\varDelta G=\varDelta H-T\cdot \varDelta S$$

The values of the relevant significant thermodynamic parameters ∆H, ∆S and ∆G are presented in Table 4.

**Table 4:** The thermodynamic parameter values for Pb(II) adsorption by JPB.

T (K)	∆H (kJ·mol^−1^)	∆S (J·mol^−1^·K^−1^))	∆G (kJ·mol^−1^)
298.0			−28.99
303.0	22.52	172.8	−29.85
308.0			−30.72

Because ∆H was positive, the adsorption process was endothermic and the adsorption would be stimulated at a higher temperature to a certain extent^43^; furthermore, ∆S with positive value reflected the random and disordered nature of the solid-solution interface during Pb(II) adsorption by JPB^30^. Moreover, the values of ∆G for different temperatures were negative, which indicated that the adsorption experiment process could take place spontaneously^30^, and meanwhile, the negative values of ∆G were decreased with the solution temperature increasing, which showed that the process of lead ion adsorption in JPB was stimulated at elevated temperatures^44,45^.

### Desorption and reusability

**Figure 14:**
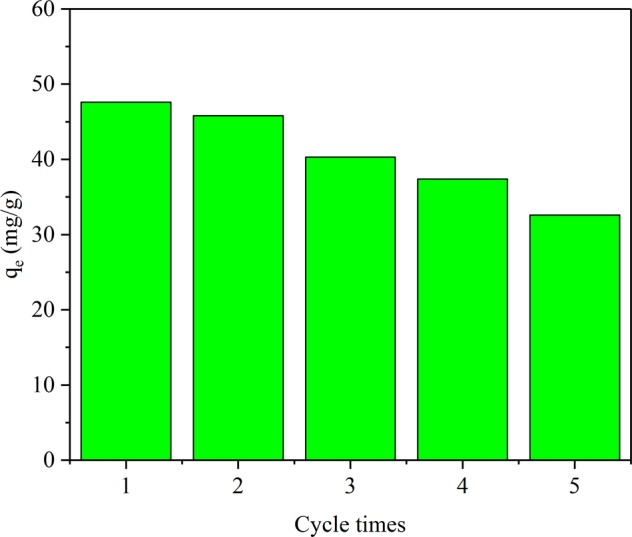
Adsorption capacity of Pb(II) by JPB for various recycling times.

A reusable adsorbent could considerably decrease the operational cost of the purification of metal ions^27^. In the desorption and reusability test experiments, and the JPB was utilized five times. The adsorption capacity for each time is shown in Fig. 14., and it was obvious that the adsorption capacity of JPB still reached up to 70% of its initial adsorption capacity after five recycles, which illustrated that the recycled JPB adsorbent still possessed a relatively considerable adsorption capacity for Pb(II). Moreover, the adsorbed Pb(II) could be recycled as a precious metal resource. Hence, this JPB adsorbent, with properties of high efficiency and recyclability, is noteworthy for practical applications in lead ion removal and recovery from wastewater.

## Conclusion

In this work, jujube pit biochar (JPB) was prepared using a simple and low-cost method, which could be used as an efficient material for lead ion removal. The nitrogen adsorption-desorption test results suggested that the JPB had large surface area, and under optimal conditions, the maximum adsorption amount of JPB for lead ions reached 137.1 mg/g. Several adsorption behaviors were studied based on parameters in the adsorption process. And a significant improvement on adsorption capacity was determined by increasing the pH, and the influence of coexisting ions, such as K^+^ and Na^+^, was quite weak on the adsorption capacity of JPB. Moreover, the uptake capacity could reach equilibrium in 30 min. Furthermore, the adsorption capacity of JPB still reached up to 70% of its initial adsorption capacity after five rounds of recycling, and the higher temperature could make a promotability for adsorption capacity. The study results demonstrated that JPB prepared from jujube pit possessed excellent adsorption ability for Pb(II) ions and had great potential for actual applications.
